# Efficacy analysis of robot-assisted percutaneous kyphoplasty in the treatment of osteoporotic vertebral compression fractures

**DOI:** 10.3389/fsurg.2026.1773066

**Published:** 2026-06-22

**Authors:** Xi-Fang Wei, Wen-Bo Wei, Jian-Zhang Jia

**Affiliations:** 1 Health Center, People's Hospital of Tongchuan, Tongchuan, Shaanxi, China; 2 State Key Laboratory for Manufacturing Systems Engineering, Xi'an Jiaotong University, Xi'an, Shaanxi, China; 3 Department of Orthopedics, Shaanxi Provincial People's Hospital, Xi'an, Shaanxi, China; 4 Department of Pain, People's Hospital of Tongchuan, Tongchuan, Shaanxi, China

**Keywords:** osteoporotic vertebral compression fracture (OVCF), pain, percutaneous kyphoplasty (PKP), robot, VAS

## Abstract

**Background:**

This study retrospectively evaluates the effectiveness of robot-assisted percutaneous kyphoplasty (PKP) in the treatment of osteoporotic vertebral compression fractures (OVCF).

**Methods:**

Between October 2022 and October 2023, a total of 92 hospitalized OVCF patients at Shaanxi Provincial People's Hospital were divided into two groups: the traditional group (*n* = 45, PKP) and the robot-assisted group (*n* = 47, robot-assisted PKP). Both groups were followed up for one year after surgery. The study compared surgical outcomes, VAS scores, vertebral body height, Cobb angle, ODI scores, and the rate of cement leakage between the two groups.

**Results:**

Compared to the traditional group, the robot-assisted group had fewer fluoroscopy frequency (11.25 ± 2.27 vs. 14.58 ± 3.63), fewer puncture needle adjustments (2.03 ± 0.25 vs. 5.51 ± 1.14), and a shorter operative time (36.53 ± 3.74 min vs. 42.25 ± 2.59 min, *P* < 0.05). However, the robot-assisted group needed longer preparation time (40.16 ± 2.57 min) than the traditional group (8.18 ± 1.03 min) (*P* < 0.05). VAS scores in both groups were reduced at 1 day, 3 months, 6 months and 12 months compared to preoperative levels. At 6 months postoperatively, both groups had higher vertebral body midline height and lower Cobb angle (*P* < 0.05). However, we found no significant differences between the two groups (*P* > 0.05). Notably, the incidence of cement leakage was significantly lower in the robot-assisted group (*P* < 0.05).

**Conclusion:**

Robot-assisted PKP can reduce fluoroscopy exposure and bone cement leakage, and have a shorter surgery time.

## Introduction

Osteoporotic vertebral compression fracture (OVCF) is a common complication of osteoporosis in middle-aged and elderly individuals (1). It can cause lower back pain, limited mobility, and in severe cases, it may lead to kyphosis and neurological symptoms in the lower limbs (2, 3). Percutaneous Kyphoplasty (PKP) is an interventional procedure for the treatment of OVCF. The surgeon injects special bone cement into the vertebral body, which can provide structural reinforcement, improve spinal function, and alleviate patients' pain (4). However, PKP has several problems. 1. Differences in soft tissue thickness and pedicle anatomy make it difficult to determine the puncture site and trajectory. Multiple puncture attempts may damage the facet joints, causing persistent postoperative pain. At the same time, it may increase the risk of cement leakage and compromise recovery. 2. x-ray fluoroscopy during the surgery increases the surgeon's risk of radiation-induced malignancies. 3. Insufficient distribution of cement can affect stability and cause secondary fracture (5, 6). How to reduce these problems is currently a hot topic in clinical research.

Recently, the application of surgical robots in orthopedic surgery for improving precision in punctures, reduce fluoroscopy times, customize preoperative and intraoperative planning options, and improve ergonomics for surgeons (7, 8). Robot-assisted PKP may help decrease number of fluoroscopy frequency, minimize bone cement leakage, and lower the incidence of postoperative residual pain. In this study, we compared the clinical use and outcomes of traditional PKP, robot-assisted PKP in patients with OVCF, aiming to evaluate the effectiveness of robot-assisted PKP in the treatment.

## Methods

### General data

This retrospective review included 92 OVCF patients treated at Shaanxi Provincial People's Hospital from October 2022 to October 2023. The study was approved by the Clinical Research Ethics Committee of Shaanxi Provincial People's Hospital (Approval No. 2023-021) and adhered to the guidelines of Good Clinical Practice, as well as the principles outlined in the Helsinki Declaration.

The patients were divided into two groups: a traditional group (45 patients, PKP surgery) and a robot-assisted group (47 patients, robot-assisted PKP surgery). The advantages, disadvantages, and potential complications of both approaches were fully explained to the patients before surgery, and the patients then selected their preferred approach. In the traditional group, there were 18 males and 27 females. The time from fracture to surgery ranged from 2 to 21 days, with (10.51 ± 3.23) days. The patients' ages ranged from 63 to 78 years, with (67.80 ± 4.29) years. The body mass index (BMI) ranged from 19.9 to 26.3 kg/m², with (23.25 ± 1.36) kg/m². The affected vertebral segments included 7 cases of T11, 18 cases of T12, 9 cases of L1, 6 cases of L2, and 5 cases of L3. According to the Genant's semi-quantitative assessment (9), the severity of vertebral compression was classified as mild in 11 cases, moderate in 24 cases, and severe in 10 cases. In the robot-assisted group, there were 21 males and 26 females. The time from fracture to surgery ranged from 2 to 18 days, with (10.05 ± 3.26) days. The patients' ages ranged from 61 to 78 years, with (68.25 ± 4.11) years. The BMI ranged from 20.1 to 27.8 kg/m², with (23.41 ± 1.40) kg/m². The affected vertebral segments included 10 cases of T11, 14 cases of T12, 13 cases of L1, 7 cases of L2, and 4 cases of L3. Vertebral compression severity was classified as mild in 16 cases, moderate in 21 cases, and severe in 10 cases. There were no statistically significant differences in baseline characteristics between the two groups (*P* > 0.05), indicating comparability.

### Inclusion and exclusion criteria

Inclusion Criteria: 1) single-level OVCFs, 2) older than 60 years, 3) low back pain that affects patients' quality of life and is ineffective to medical therapy, 4) bone mineral density (BMD) T-scores less than −2.5, 5) MRI showed fresh vertebral fracture (low T1 and high T2 signals), with an intact posterior wall, and no intraspinal space-occupying lesion or spinal cord compression, 6) patients willing to receive PKP treatment.

Exclusion Criteria: 1) infection, 2) radicular and/or cord compression syndrome, 3) patients who are unable to undergo operation due to mental or organ dysfunction, 4) burst vertebral fracture with spinal canal stenosis and neurologic deficit, 5) spinal infection or skin disease, 6) previous lumbar surgery, 7) secondary osteoporosis or pathological fractures, 8) Postoperative follow-up less than 1 year.

### Treatment methods

Prior to surgery, the surgeon explained the advantages, disadvantages, and potential complications of both surgical methods to the patients, who then chose the preferred approach. All surgeries were performed by two surgeons, each with over 10 years of experience in spine surgery. Both groups received local anesthesia and underwent bilateral pedicle punctures in the prone position. The surgery team followed standard procedures by draping and disinfecting the operation area.

Traditional Group (PKP): The injured target vertebra was identified and marked under fluoroscopic guidance using a C-arm system. The surgeon administered local anesthesia at the marked site, and punctured at 2 o'clock position on the right pedicle and the 11 o'clock position on the left. A puncture needle was inserted from the pedicle into the posterior a half of the vertebral body under fluoroscopic guidance. Removed the puncture needle core and inserted the working cannula. The reamer bit was implanted and drilled to 3 mm from the anterior edge of the vertebral body. After withdrawing the drill, a balloon dilator was implanted into the anterior half of the vertebral body, and contrast medium was injected into the balloon under intermittent fluoroscopic monitoring. The developer was withdrawn, and then the balloon was removed. Bone cement was injected bilaterally under fluoroscopic monitoring. Once the cement hardened and the vertebral body restored its height, the working cannula was removed.

**Figure 1:**
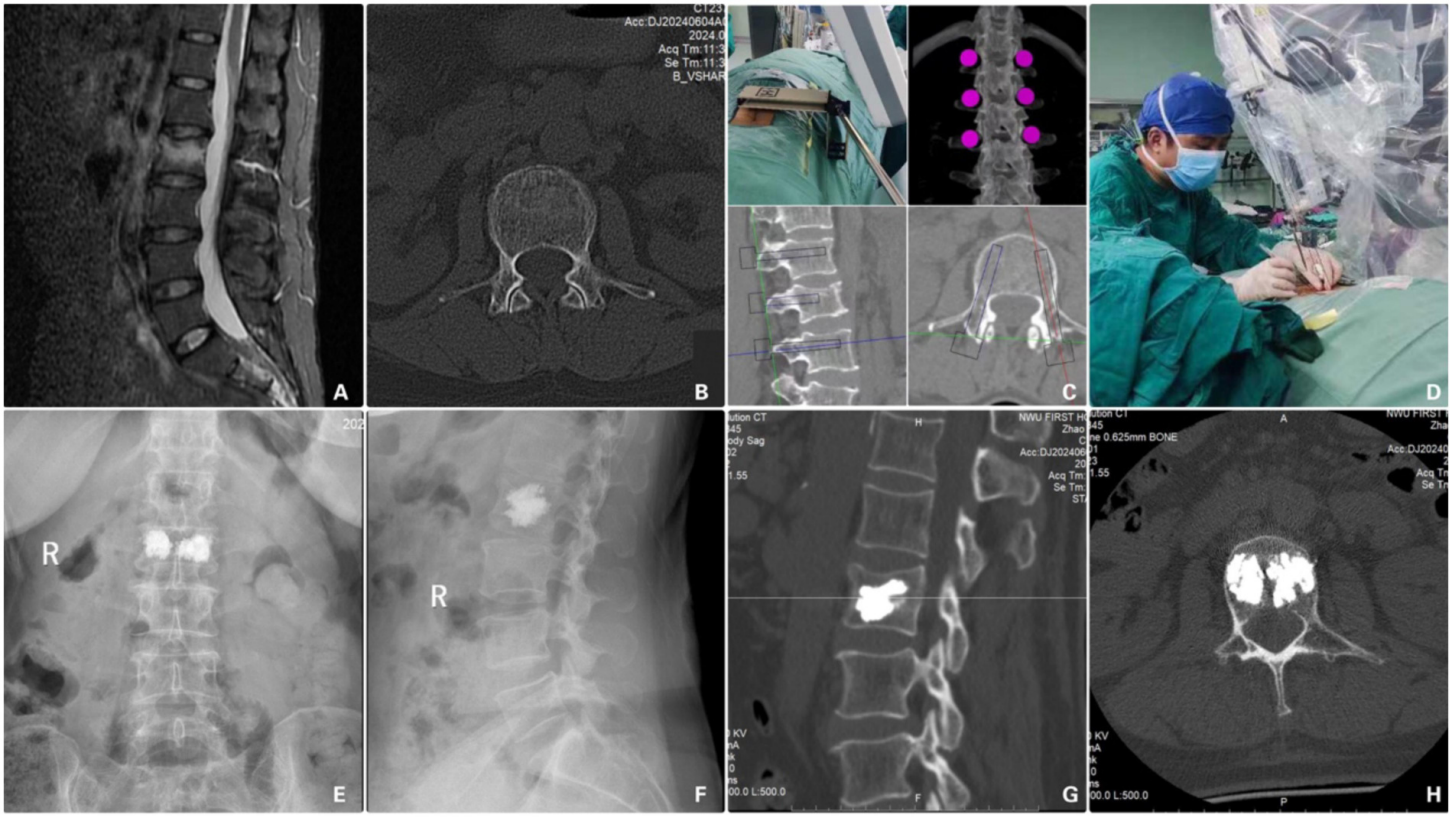
**(A)** preoperative MRI Fat-suppressed sequence, **(B)** preoperative axial CT scan, **(C)** intraoperative robot-assisted planning, **(D)** intraoperative robot-guided operation, **(E,F)** x-ray images on postoperative Day 2, **(G,H)** CT images on postoperative Day 2.

Robot-assisted Group (Robot-Assisted PKP): The CT images of the fractured vertebra were transmitted to the ORTHBOT spinal robotic workstation (ORTHBOT, Shenzhen Xijunte Medical Technology Co., Ltd.), for preoperative puncture path planning. First, the tracer was fixed, and a sterile protective sleeve was used to isolate the robot arm. The robot arm automatically adjusted the posture and the end position. After accurate positioning by the robot, the working sleeve was placed. Under C-arm guidance, the robotic arm was then positioned at the planned entry points, and the puncture needles were placed accordingly. Bilateral punctures were performed by the robotic arm (Figures 1C,D), with real-time monitoring of pressure transmission and simulated trajectory. Once the puncture needles were confirmed to be correctly positioned, the working cannula was inserted. The follow-up operation was the same as that of the Traditional Group.

After surgery, both groups received anti-osteoporosis treatments. Patients were required to wear a brace for early mobilization and rehabilitation exercises.

### Data collection

The basic characteristics of the OVCFs patients were recorded, including age, gender, body mass index (BMI), T value of bone mineral density (BMD) and vertebral fracture distribution.

During the surgery, the fluoroscopy frequency and operative duration were recorded. The lengths of stay of patients were also assessed. Postoperative complications, including cement leakage, infection and thrombosis, were recorded. The midline height of the fractured vertebra and Cobb angle were measured using x-ray imaging before surgery and at 6 months postoperatively.

To evaluate the recovery of the patients, the pain conditions caused by vertebral fracture were measured by the Visual Analog Scale (VAS), and the recovery of vertebral function was measured by the Oswestry Disability Index (ODI). VAS and ODI scores were recorded at the time points of before the surgery and 1 day and 3, 6, 12 months after the surgery.

### Statistical analysis

Statistical analysis was performed using SPSS 25.0. Measurement data were expressed as mean ± SD and analyzed using the t-test. Repeated measures analysis of variance (ANOVA) was used for comparisons involving multiple time points of a single variable. Categorical data were expressed as percentages and analyzed using the chi-square (*χ*²) test. Effect sizes were calculated to assess the magnitude of differences, with Cohen's d reported for *t*-tests. A *P*-value < 0.05 was considered statistically significant.

## Results

### Surgical outcomes

The robot-assisted group required fewer puncture needle adjustments and fluoroscopy frequency compared to the traditional group, and the operative time was significantly shorter (*P* < 0.05). Hospitalization cost was higher in the robot-assisted group. (Table 1).

**Table 1:** Comparison of general data between two group.

Characteristic		Group traditional (*n* = 45)	Group robot-assisted (*n* = 47)	t/(x^2^)	*P*
Male/female		18/27	21/26	0.206	0.650
Age (years)		67.80 ± 4.29	68.25 ± 4.11	−0.207	0.613
BMI (kg/m2)		23.25 ± 1.36	23.41 ± 1.40	−0.105	0.382
Spine levels	T11	7	10	1.825	0.77
	T12	18	14		
	L1	9	13		
	L2	6	6		
	L3	5	4		
Genant semi-quantitative grading	Mild	11	16	1.083	0.582
	Moderate	24	21		
	Severe	10	10		
Time from fracture to surgery (days)	10.51 ± 3.23	10.05 ± 3.26	0.142	0.857	
Needle Adjustments (times)	5.51 ± 1.14	2.03 ± 0.25	29.215	＜0.001	
Operative time (mins)	42.25 ± 2.59	36.53 ± 3.74	12.320	＜0.001	
Preparation time (mins)	8.18 ± 1.03	40.16 ± 2.57	11.263	＜0.001	
Fluoroscopy frequency (times)	14.58 ± 3.63	11.25 ± 2.27	7.621	＜0.001	
Hospitalization cost (yuan)	8,671 ± 279	12,523 ± 518	17.53	＜0.001	

Numeric data were expressed as Mean ± SD and analyzed by Independent-Samples *T*-test. Categorical data were expressed by the number of patients (%) and were analyzed with the *χ*^2^ test. *P < 0.05, Group traditional vs. Group robot-assisted.

BMI, body mass index.

### Comparison of VAS

At postoperative day 1, 3, 6 and 12 months, the VAS scores in both groups were significantly lower than preoperative levels (*P* < 0.05). However, there was no statistically significant difference in VAS scores between the two groups (*P* > 0.05). (Table 2).

**Table 2:** VAS scores ($\bar{x}\pm s$, points).

Group	Cases	Pre-operation	Post-operation Day 1	Post-operation Month	Post-operation 6 Months	Post-operation 12 Months
Traditional	45	7.67 ± 0.52	3.24 ± 1.13*	2.17 ± 0.93*	1.41 ± 0.49*	1.30 ± 0.41*
Robot-assisted	47	7.73 ± 0.68	3.32 ± 1.26*	2.01 ± 1.01*	1.29 ± 0.46*	1.27 ± 0.35*
Time F, P		815.174, ＜0.001				
Group F, P		0.216, 0.614				
Time * Group F, P		0.918, 0.219				

Data are presented as mean ± SD. The groups were compared by repeated measures analysis of variance (ANOVA). Bonferroni correction was used to correct multiple comparisons. vs. pre-operation in the same group, *P < 0.05. .

VAS, visual analog scale.

### Comparison of ODI

At postoperative day 1, 3, 6 and 12 months, the ODI scores in both groups were significantly lower than preoperative levels (*P* < 0.05). However, there was no statistically significant difference in ODI scores between the two groups (*P* > 0.05). (Table 3).

**Table 3:** ODI scores ($\bar{x}\pm s$, points).

Group	Cases	Pre-operation	Post-operation Day 1	Post-operation 3 Month	Post-operation 6 Months	Post-operation 12 Months
Traditional	45	81.21 ± 1.17	40.39 ± 1.08*	38.39 ± 0.91*	32.39 ± 0.98*	30.14 ± 0.82*
Robot-assisted	47	80.10 ± 1.32	40.10 ± 1.12*	37.19 ± 0.84*	31.19 ± 1.15*	30.55 ± 0.74*
Time F, P		1,944.615，＜0.001				
Group F, P		0.175，0.628				
Time * Group F, P		0.915，0.382				

Data are presented as mean ± SD. The groups were compared by repeated measures analysis of variance (ANOVA). Bonferroni correction was used to correct multiple comparisons. vs. pre-operation in the same group, *P < 0.05.

ODI, oswestry disability index.

### Vertebral height and cobb angle

At 6 months postoperatively, the vertebral midline height in both groups was significantly higher than preoperative values, while the Cobb angle was significantly reduced (*P* < 0.05). However, there was no statistically significant difference between the two groups (*P* > 0.05). (Table 4).

**Table 4:** Vertebral height and cobb angle $\left(\bar{x}\pm s\right)$.

Group	Cases	Vertebral midline height (mm)		Vertebral cobb angle (°)	
		Pre-operation	Post-operation 6 Months	Pre-operation	Post-operation 6 Months
Traditional	45	17.38 ± 3.24	26.55 ± 3.74*	38.59 ± 5.56	14.26 ± 2.98*
Robot-assisted	47	17.24 ± 3.42	27.05 ± 2.75*	38.45 ± 6.51	14.55 ± 2.23*
t		0.291	1.055	0.160	0.763
P		0.771	0.293	0.873	0.446

Numeric data were expressed as Mean ± SD and analyzed by Independent-Samples *T*-test. *P < 0.05, Group traditional vs. Group robot-assisted.

### Cement leakage

Cement leakage occurred in 14 cases (40.62%) in the control group and 8 cases (14.58%) in the observation group. The leakage rate in the robot-assisted group was lower than in the control group (*χ*² = 16.289, *P* < 0.05).

## Discussion

For OVCFs patients with severe pain and ineffective conservative treatment, surgery is an option (1). However, many OVCF patients are elderly, have weakened organ function, or suffer from conditions like hypertension, hyperglycemia, and coronary heart disease (1). This situation makes general anesthesia and open surgery more difficult. PKP becomes a preferred option due to its minimal invasiveness, use of local anesthesia, and quick recovery (10). However, there is a risk of bone cement leakage after PKP due to the difficulty of manual puncture. Bone cement leakage can cause neurological dysfunction or pulmonary embolism, affecting postoperative recovery and patient quality of life (11, 12). With the development of robotic-assisted technology, puncture accuracy is significantly higher than manual puncture (13). In this study, we explored whether robot-assisted PKP reduces bone cement leakage by enhancing accuracy. We compared robot-assisted PKP and traditional PKP in OVCF patients, assessing treatment efficacy and leakage rates. Results showed the robot-assisted group required fewer needle adjustments and reduced fluoroscopy usage. Thus, Robot-Assisted PKP offers promising preliminary evidence supporting the advantages in reducing operative time, and cement leakage.

The leakage rate of bone cement after PKP is about 7%–50% (13, 14). Bone cement leakage can cause nerve damage and pulmonary embolism, leading to serious consequences (15). Therefore, how to reduce the occurrence of bone cement leakage has become a focus of attention for doctors. Severe compression fractures and an incomplete posterior vertebral wall are associated with a higher risk of bone cement leakage; therefore, caution should be exercised when performing PKP. Genant's semi-quantitative assessment (9) is used to evaluate patients with vertebral compression fractures, aiding in determining their suitability for PKP and predicting the risk of cement leakage. Consequently, in our study, patients with severe compression fractures were less frequently selected for this procedure. The cement leakage during PKP is related to the timing of polymethylmethacrylate (PMMA) injection and the position of the puncture needle (11). The PMMA injected during adhesion period has a higher diffusion rate. It tends to spread along bone trabecula and fracture gaps, increasing the risk of cement leakage (16). The puncture needle's position also affects leakage. If placed in the fracture gap, the PMMA easily spreads along the fracture gap, leading to leakage (17). Preoperative CT scans help identify fracture lines and guide puncture planning, reducing cement leakage risk (18). However, PVP is performed under local anesthesia, and patients remain awake and active. During the puncture process, surgeons need to identify bony structures by moving the needle tip and adjust the puncture route multiple times. Due to pain, the patient's intraoperative position may change, resulting in deviations from the preoperative plan and causing bone cement leakage. Our research found that the incidence of bone cement in the traditional group was significantly higher than that in the robot-assisted group, and the leakage rate was similar to that reported in the literature (13).

Surgeons must adjust the puncture needle continuously using fluoroscopy. Frequent adjustments reduce accuracy, damage joint surfaces, and may cause local hematoma and severe pain (19). Additionally, repeated fluoroscopy exposes surgeons to high radiation doses, increasing the risk of tumors (20). Reducing intraoperative fluoroscopy can avoid local pain for patients and the risk of cancer for surgeons. Through preoperative imaging planning, the robot can clearly identify vertebral tissues and soft tissue, then automatically reaches the puncture point using preset parameters.

Robots have high precision and good stability, which can avoid the inaccuracy and fatigue of manual operation (21). This allows for fast, accurate, one-time puncture insertion, which can reduce damage to the articular process and joint capsule, and minimize postoperative pain. In addition, the robot system we used can detect pressure changes during the puncture process. If there is an abnormal pressure change, the robot can stop the operation in time. In robot-assisted PVP, the puncture is performed along a preset trajectory. This eliminates the need for repeated fluoroscopy, reducing the number of fluoroscopic exams and shortening the operation time (22). Although the preparation time before puncture was longer than in the control group, which is related to robot preparation, startup registration, and planning of surgical approaches. But patients will not increase the risk of surgery due to prolonged prone position. However, this preparation time can significantly reduce surgical time, intraoperative fluoroscopy frequency, and lower the risk of bone cement infiltration and joint process injury during surgery. Our results show that the cost of robot-assisted surgery is higher than that of traditional surgery, which is associated with longer operating room occupancy time and additional charges for robot-navigated surgery. However, with medical insurance coverage, the patient's final financial responsibility does not exceed 1,000 yuan.

The results of this study showed that the VAS scores for both groups were lower at each postoperative time point compared to preoperative scores. The midline height, Cobb angle, and ODI of the injured vertebrae improved 6 months after surgery, but there was no significant difference between the groups. However, this study has certain limitations. The study design was nonrandomized, which may have introduced sampling bias because it was a single-center retrospective analysis with a relatively small sample size. Future multi-center prospective studies with longer follow-up are needed to verify the efficacy of robot-assisted PVP for OVCF treatment.

## Conclusion

In summary, patients treated with PKP or robot-assisted PKP can restore vertebral height and Cobb angle. Both methods relieve pain and improve lumbar function. Compared with PKP, robot-assisted PKP requires fewer needle adjustments and fluoroscopy frequency. It also has shorter operation time, and results in a lower incidence of bone cement leakage.

## Data Availability

The raw data supporting the conclusions of this article will be made available by the authors, without undue reservation.
